# Multiple innate antibacterial immune defense elements are correlated in diverse ungulate species

**DOI:** 10.1371/journal.pone.0225579

**Published:** 2019-11-27

**Authors:** Brian S. Dugovich, Lucie L. Crane, Benji B. Alcantar, Brianna R. Beechler, Brian P. Dolan, Anna E. Jolles

**Affiliations:** 1 Department of Integrative Biology, Oregon State University, Corvallis, OR, United States of America; 2 Department of Biomedical Sciences, Oregon State University, Corvallis, OR, United States of America; 3 Wildlife Safari, Winston, OR, United States of America; Universitat Autonoma de Barcelona, SPAIN

## Abstract

In this study, we aimed to evaluate to what extent different assays of innate immunity reveal similar patterns of variation across ungulate species. We compared several measures of innate antibacterial immune function across seven different ungulate species using blood samples obtained from captive animals maintained in a zoological park. We measured mRNA expression of two receptors involved in innate pathogen detection, toll-like receptors 2 and 5 (TLR2 and 5), the bactericidal capacity of plasma, as well as the number of neutrophils and lymphocytes. Species examined included aoudad (*Ammotragus lervia*), American bison (*Bison bison bison*), yak (*Bos grunniens*), Roosevelt elk (*Cervus canadensis roosevelti*), fallow deer (*Dama dama*), sika deer (*Cervus nippon*), and Damara zebra (*Equus quagga burchellii*). Innate immunity varied among ungulate species. However, we detected strong, positive correlations between the different measures of innate immunity–specifically, TLR2 and TLR5 were correlated, and the neutrophil to lymphocyte ratio was positively associated with TLR2, TLR5, and bacterial killing ability. Our results suggest that ecoimmunological study results may be quite robust to the choice of assays, at least for antibacterial innate immunity; and that, despite the complexity of the immune system, important sources of variation in immunity in natural populations may be discoverable with comparatively simple tools.

## Introduction

Innate immune responses are important both for rapidly marshaling the body’s defenses following pathogen exposure and for initiating adaptive immune responses which can help confer long-term protection [1]. There are many components of the vertebrate innate immune system that can be targeted for study, including recognition elements, effector proteins, and cells. Pathogen recognition receptors (PRRs) recognize conserved microbial components, such as proteins, lipids, or nucleic acids, and several cell types express PRRs, such as toll-like receptors (TLRs), Nod-like receptors, and RIG-I (retinoic acid-inducible gene I) like receptors [2]. Engagement of PRRs with their appropriate ligands leads to cellular signaling events that can drive immune responses [3]. The resulting inflammatory response can induce the production of many components, such as acute phase proteins and proteins of the complement pathway, which have antimicrobial properties and are part of the innate immune response [4]. Cellular signaling during the innate immune response can also serve to recruit particular leukocytes, such as neutrophils and macrophages, which are known to phagocytose pathogens [5]. Neutrophils have potent antibacterial capabilities when activated. Thus, multiple immune parameters are available for measuring innate immune defenses. As such, for ecoimmunological studies, it is important to evaluate to what extent the choice of immune marker is likely to alter study conclusions and to avoid redundancy between assays.

Assay choice for ecoimmunological studies is fraught with limitations related to multiple factors including, but not limited to, the practicality of working in field environments, the lack of species specific or validated cross-reactive reagents, and cost. Therefore, choosing the appropriate immunological measures to evaluate immune mechanisms in a study system is the most basic question to studying wildlife ecoimmunology [6]. However, the ideal immunological assay strategy for a laboratory setting is often not feasible in the field due to the lack of mobile equipment and an appropriate lab space. Additionally, small amounts of sample material, such as blood, often limit the number and types of assays which can be performed. A successful wildlife ecoimmunology study depends on efficiency and limiting the ecoimmunology toolkit to the minimum assays required to answer the research question in mind.

Innate immune defenses are frequently evaluated in ecoimmunological studies, due to their importance as the immediate defense against pathogen invasion [7]. In this report we used multiple measures to assess innate antibacterial immune defenses from a single blood sample in seven wild ungulate species maintained in a zoological park. Our ecoimmunology toolkit to measure innate immune defenses included expression of toll-like receptor 2 (TLR2) and toll-like receptor 5 (TLR5) in leukocytes, determining the ratio of neutrophils to lymphocytes (calculated from absolute numbers of neutrophils and lymphocytes in blood samples), and measuring the bactericidal abilities of plasma using a bacterial killing assay (BKA). TLR2 is a PRR which, in combination with either TLR1 or TLR6, recognizes many bacterial-derived products such as lipoproteins and peptidoglycan [8] which are present in most bacterial species at varying levels. TLR5 is a PRR which recognizes bacterial flagella [9], which are expressed by nearly all bacteria. Neutrophils are one class of leukocyte that are present in high abundance and have potent antibacterial capabilities when activated. The ratio of neutrophils to lymphocytes (NLR) is an important clinical measure of inflammatory status [10] and physiological stress [11], and normal ranges vary between species [12]. Increased NLR is an indication of inflammation, which may also increase those plasma components that have antibacterial properties. A bacterial killing assay (BKA) directly measures the ability of plasma to kill a laboratory strain of bacteria and has been used as a proxy for innate immune competence [13]. In whole blood, leukocytes, such as macrophages and neutrophils, kill bacteria, but the mechanism that allows plasma to kill bacteria is less clear. The complement system is likely involved [14,15], as heat inactivation of plasma showed decreased bactericidal activity [16]. However, several other antibacterial proteins and peptides are present in blood (e.g. cathelicidins, defensins) which may also contribute to the total antibacterial innate immune response [17,18].

Ungulates provide a tractable, ecologically and economically important taxon to investigate questions in ecoimmunology and disease ecology [19,20] since ungulates commonly interface with domestic livestock resulting in infectious disease spillover [21]. Despite their ubiquity, wild ungulates are a clade of animals whose immune functions have been relatively understudied. One factor complicating the study of wild ungulate immune defenses, is that immobilization drugs and capture stress can alter some blood test results [22,23], while some immunological assays in other animals are robust to capture techniques [24]. The sensitivity of some immunological assays to animal capture and handling might obscure patterns of variation due to ecological drivers that are of interest in ecoimmunological studies. However, in this study, we were not attempting to identify drivers of the immune defense; instead, we focused on testing whether different assays for antibacterial innate immunity are likely to yield congruent patterns. Thus, we hypothesized that TLRs, NLR, and BKA would correlate within individual animals and across ungulate species, indicating that the choice of innate immunological assay should not affect the patterns detected in ecoimmunological studies qualitatively.

## Materials and methods

### Sample collection

Blood was collected from captive adult ungulates at Wildlife Safari in Winston, Oregon in collaboration with the park and veterinary staff. Table 1 lists the animal species studied. Immobilizations were performed as early in the morning as possible to minimize the risk of hyperthermia. The animal was immobilized with an air rifle dart containing a mixture of drugs delivered intramuscularly. The anesthesia protocol was tailored to each species. Carfentanil or thiafentanil (Zoopharm), xylazine (VetOne), and ketamine (VetOne) were used in combination to immobilize the larger ungulates (>150 kg); while telazol (Zoetis) and xylazine were used in the smaller ungulates (<150 kg). When the animal was immobilized, a physical exam was performed, and routine veterinary care (intravenous catheter and fluids, dart wound care, and hoof trimming) was provided. Heart rate, respiratory rate, and temperature were monitored at 5-minute intervals for the length of the sedation (approximately 20 minutes in most animals). Time to blood sampling ranged from 5–20 minutes. Blood samples (approximately 5 ml) were collected from jugular or lateral saphenous veins and placed in heparinized tubes. Time of blood draw to blood testing varied from 30 minutes to 2.5 hours. For reversal, naltrexone (Zoopharm) was administered intravenously, and atipamezole (Zoetis) was administered intramuscularly. Animals were monitored during the time immediately following recovery and then periodically throughout the rest of the day. Plasma was isolated in heparinized tubes by centrifugation in a Vetlab Combispin Centrifuge, and 500 μl aliquots of plasma were stored at -80°C.

**Table 1:** Adult animals included in the study. The following animals were immobilized and sampled at Wildlife Safari (2013–2014).

Common Name	Scientific Name	Family	Number in study (females, males)
Aoudad	*Ammotragus lervia*	Bovidae	3(2,1)
Bison	*Bison bison bison*	Bovidae	3(3,0)
Yak	*Bos grunniens*	Bovidae	3(3,0)
Roosevelt elk	*Cervus canadensis roosevelti*	Cervidae	13(8,5*)
Fallow deer	*Dama dama*	Cervidae	4(4,0)
Sika deer	*Cervus nippon*	Cervidae	15(14,1)
Damara zebra	*Equus quagga burchellii*	Equidae	3(2,1)

*includes 3 castrated males

### Leukocyte differential analysis

Total and differential white blood cell counts were used to quantify the leukocytes present in the animal’s blood. Total white blood cell count was obtained using a HemaTrue analyzer (Heska SN 61372) in Wildlife Safari’s veterinary laboratory. The cow setting was used for cervids and bovids while the horse setting was used for zebra. Differential counts were performed from a blood smear slide made on the day of capture and stained with Diff-Quik. Absolute numbers of each leukocyte type were determined by multiplying the total white blood cell count by the differential percentages.

### Bacterial killing assay

The bacterial killing assay (BKA) has been modified from techniques introduced by Tieleman et al. [25] and French & Neuman-Lee [26], but rather than measure bacteria concentration at a selected endpoint, the time to the exponential growth phase was measured, which is determined by the initial concentration of bacteria in the solution. The DH5α laboratory strain of *Escherichia coli* containing the gentamycin resistance plasmid pJN105 [27] was used as a target for killing by plasma components using a previously established protocol [16,28]. Briefly, 5 colonies from an agar plate were collected using a BBL^™^ prompt and diluted in sterile phosphate buffered saline, and 30 μl (approximately 20,000 CFU) was distributed to each well of a 96-well plate. Frozen plasma samples were thawed for the first time in an ice water bath and were diluted 1:75 in PBS, and 50 μl was mixed with the bacterial solution in the plate wells and incubated for 30 minutes at 37°C. Tryptic soy broth (100 μl, with 10 μg/ml gentamycin) was then added to the wells, and the plate was incubated with gentle agitation at 37°C. Starting at 5 hours, and every hour thereafter, the absorbance of each well at 600 nm was recorded and plotted as a function of time. Each sample was run in triplicate on a different plate, and the three plates were run at the same time. The growth curve was fitted with a sigmoidal curve, using GraphPad Prism version 6.07, GraphPad Software, La Jolla, California, USA, and the time to 50% growth for each sample was determined. Additional wells lacking plasma but containing increasing dilutions of the initial starting concentration of bacteria were also included on each plate to generate a standard curve. The time to 50% growth for each standard was plotted as a function of percent dilution and fitted with a linear regression. The line of best fit was used to determine the percent reduction of bacteria in each sample (i.e., the longer the time to 50% growth, the more efficiently plasma was killing bacteria). The triplicate results were averaged to calculate the percent-reduction in bacteria. If no growth was observed, then the result was deemed 100% reduction in bacterial growth.

### RNA isolation and cDNA synthesis

Leukocytes from ~ 5 ml of whole blood collected in heparinized tubes were isolated by centrifuging blood collection tubes for 20 minutes at 1,500 RCF. The buffy coat was gently removed using a disposable transfer pipet, and contaminating erythrocytes were eliminated by lysing in ammonium-chloride-potassium lysing buffer. Leukocytes were washed in PBS and pelleted. Total RNA was extracted using Macherey-Nagel NucleoSpin RNA isolation kits following the manufacturer’s recommended guidelines. To generate cDNA, 5 μl of RNA was combined with 15 μl water and added to an RNA to cDNA EcoDry Premix reaction tube containing oligo dT primers (Clontech) following the manufacturer's recommended guidelines.

### Primer design

To design primers to work on a variety of ungulate species, most of which do not have complete genomes available, mRNA sequences from a variety of carnivores and ungulates species were aligned using Clustal W software to identify regions of homology. Universal primers for the mammalian glyceraldehyde 3-phosphate dehydrogenase (GAPDH), TLR2, and TLR5 genes were constructed using published mRNA sequences from the giant panda (*Ailuropoda melanoleuca*), horse (*Equus caballus*), cattle (*Bos taurus*), sheep (*Ovis aries*), dog *(Canis lupus familiaris*), cat (*Felis catus*), pig (*Sus scrofa*), and American bison (*Bison bison*). The mRNA sequences NM_001304846.1, NM_001163856.1, NM_001034034.2, NM_001190390.1, NM_001003142.2, NM_001009307.1, NM_001206359.1, and XM_010844969.1 were included during development for the GAPDH primer. The mRNA sequences XM_002913846.2, NM_001081796.1, NM_174197.2, NM_001048231.1, NM_001005264.3, XM_003984930.3, NM_213761.1, and XM_010840725.1 were aligned for the TLR2 primer. No mRNA sequence of the TLR5 gene was available for the horse. The mRNA sequences XM_011228904, NM_001040501.1, NM_001135926.1, NM_001197176.1, XM_011290806.1, NM_001123202.1, and XM_010852831.1 were used to construct the TLR5 primer. These mRNA sequences were uploaded from the NCBI database and aligned using ClustalW (Lasergene Software, Megalign program). The aligned sequences were reviewed, and homologous areas were investigated as possible primer sequences. The primer sequence needed to have no more than three degenerate bases and be within 250 base pairs of each other. The primer sequences and the expected length and average molecular weight of the replicated sequence are listed in the supporting information (S1 Table). Primers were purchased from IDT and validated using a random cDNA sample as a template in a polymerase chain reaction (PCR). Primers were resuspended in distilled water and 0.16 μMol of each forward and reverse primer was mixed with 1 μl cDNA, 22 μl of water, and 25 μl of 2X FideliTaq PCR Master Mix (Affymetrix) and cycled at 95°C for 15 seconds, 53°C for 15 seconds, and 68°C for 30 seconds for a total of 35 cycles. PCR products were examined by gel electrophoresis to ensure the correct size, and DNA was purified using a PCR purification kit (Macherey-Nagel) according to the manufacturer’s instructions. The concentration of the purified PCR product was determined using a Qubit 2.0 fluorometer (Invitrogen) and converted from ng/μl to copies/μl. The DNA solution was diluted in serial 10-fold dilutions and used to generate a standard curve in the subsequent quantitative PCR reactions. Our primers (sequences: S1 Table) amplified a PCR product of the predicted size in all ungulate species tested (S1 Fig) and could be used in quantitative PCR reactions.

### Quantitative PCR

Quantitative PCR was completed using Fast SYBR Green (Applied Biosciences) technology and analyzed on a StepOnePlus (Applied Biosystems) thermocycler set for fast analysis. Each PCR reaction contained 0.16 μMol of each primer, 0.5 μl of cDNA, 11.5 μl of water, and 12.5 μl 2X Fast SYBR Green master mix for a total of 25 μl. The reaction was added to a well of a 96-well plate (ABI) and cycled with a run method of one 10 minute 95°C time period and then 40 cycles of 15 second 95°C denaturing phase, 10 second 53°C annealing phase, and 30 second 72°C extension phase. A final melting curve analysis was performed for each plate to ensure that only one product was present. The cycle threshold (Ct) number for each cDNA sample was calculated using StepOne Software for StepOnePlus Real-Time PCR systems (Applied Biosystems) for all three genes. Each transcript target was run in technical triplicates and averaged. To convert Ct values to copies/μl, a standard curve was generated for each gene product and each plate run using the previously described PCR standards. TLR proteins are expressed on a variety of cell types, including leukocytes, to a varying extent. TLR2 and TLR5 copies were subsequently normalized by dividing the copies/μl for each TLR gene by the GAPDH copies/μl, and the resulting ratio was used for subsequent analysis.

### Statistical analysis

Animals captured were predominantly female (Table 1) and were mostly deemed clinically healthy by Wildlife Safari veterinary staff. Some animals immobilized for treatment of minor ailments were included in the study (S2 Table). A conservative one-way independent ANOVA power analysis showing seven groups of three samples, a large effect size of 50%, and a significance level of 0.05, showed a 23.9% chance to find significant differences confirming that we could find some strong relationships despite our small sample sizes. Non-normal distributions of the independent variables (TLR2, TLR5, plasma BKA, and NLR) were confirmed with a Shapiro-Wilk’s test, and the data were log-transformed for use in linear models. An ANOVA with Tukey’s honest significant difference (HSD) test was used to test for all pair-wise differences in immune measures between species. To confirm these differences, a permutational multivariate analysis of variance (PERMANOVA) was used to compare the immune measures to species or family. To evaluate individual level relationships between innate immune measures, linear mixed models were used. Species were used as a random effect since differences in immune measures were expected between species.

Examplemodel:Log(TLR2)∼Log(TLR5)+(1|species)

Other linear mixed models added sex as an explanatory parameter to examine if sex influenced correlations. Normality testing and Tukey’s HSD ANOVA were implemented using R version 3.4.3 (R Core Team, 2017). PERMANOVA used the vegan package [29] and used a Bonferroni correction. The lme4 package [30] used restricted maximum-likelihood (REML) for linear mixed models, and R^2^ for linear mixed models was calculated using R_Σ_^2^ [31].

## Results

### Immunological variation between ungulate species

**Fig 1:**
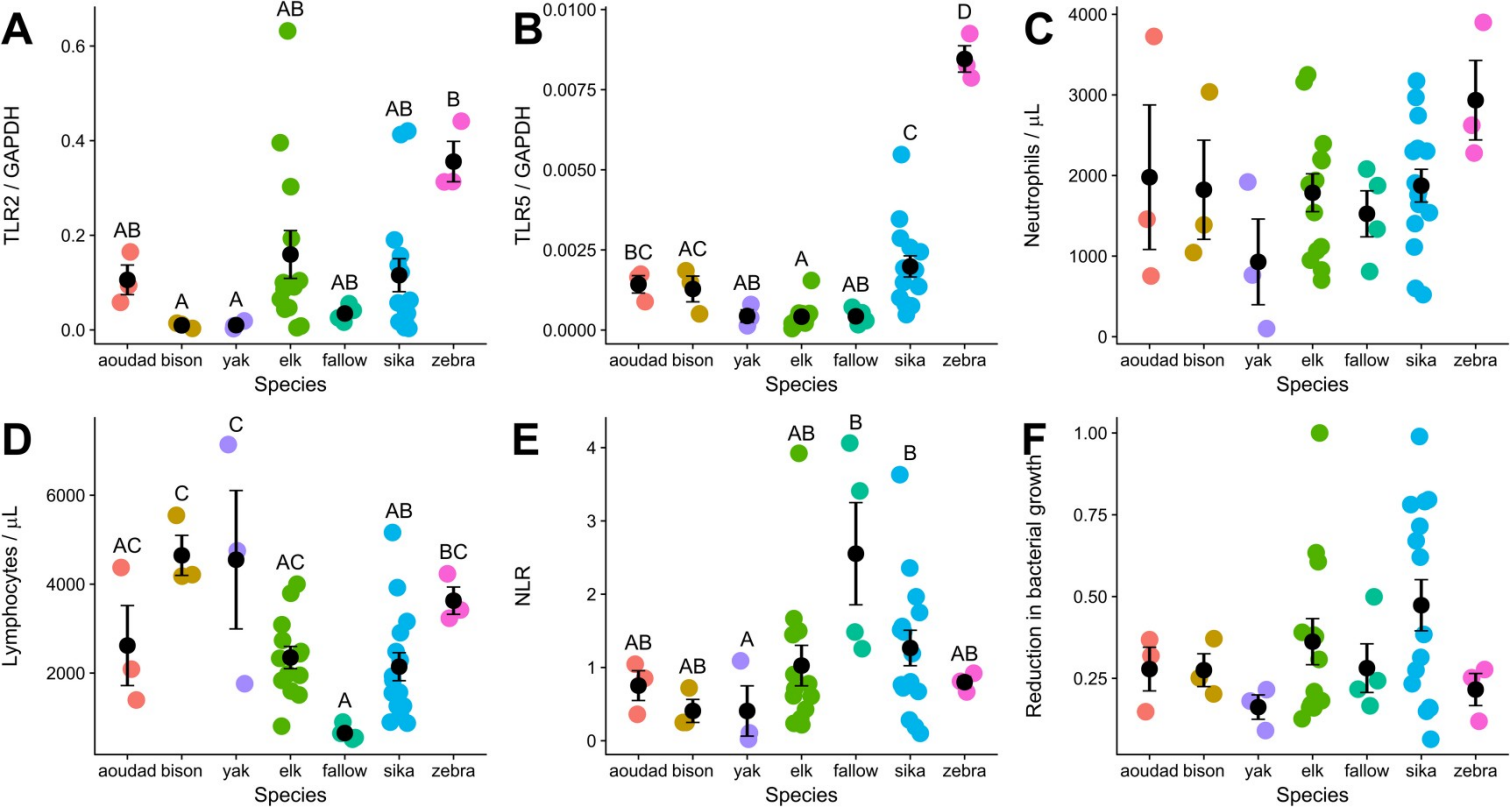
Variation in innate immune parameters among ungulate species. Each data point represents an individual animal with points color-coded by species at Wildlife Safari (2013–2014). Black points and bars represent the average and standard error for each species. Significant pairwise differences are denoted by letters above each bar. Toll-like receptor 2 (TLR2, A), Toll-like receptor 5 (TLR5, B), lymphocytes (D), and neutrophil to lymphocyte ratio (NLR) (E) were significantly different between some species. Neutrophils (C) and the bacterial killing assay (BKA) (F) were not significantly different between species.

We measured several different aspects of innate, antibacterial immune responses in ungulates and grouped the results by species to determine which, if any, parameters varied between species. Analysis of the data revealed some interesting patterns. Zebra had the highest expression of TLR2 (Fig 1A) which was significantly greater than bison and yak, though not significant when compared to other species. Zebra also expressed more TLR5 transcripts than all other ungulates (Fig 1B) and there was some variation between other species, such as sika deer expressing significantly more TLR5 than elk, yak, and fallow deer but were not statistically different when compared to aoudad and bison (Fig 1B). Absolute neutrophil counts displayed wide variation within species and between species and there were no statistically significant differences between species (Fig 1C). However, absolute lymphocyte counts did show some species to species variation with fallow deer having the fewest numbers (Fig 1D) and bison and yak with the most lymphocytes. In general, it appears that cervids tended to have a lower lymphocyte count and thus a higher NLR than the other species (Fig 1D and 1E). No difference was observed in the ability of plasma to kill bacteria between species (Fig 1F). PERMANOVA analysis of the overall suite of innate immune defenses detected significant differences between fallow deer and elk (p = 0.021) and fallow deer and sika (p = 0.021) and borderline significance between cervids and bovids (p = 0.06), but small sample sizes may have obfuscated other significant variation. Thus, some measures of innate antibacterial immune parameters differ between species, while others remain constant.

### Correlation among immune metrics

**Fig 2:**
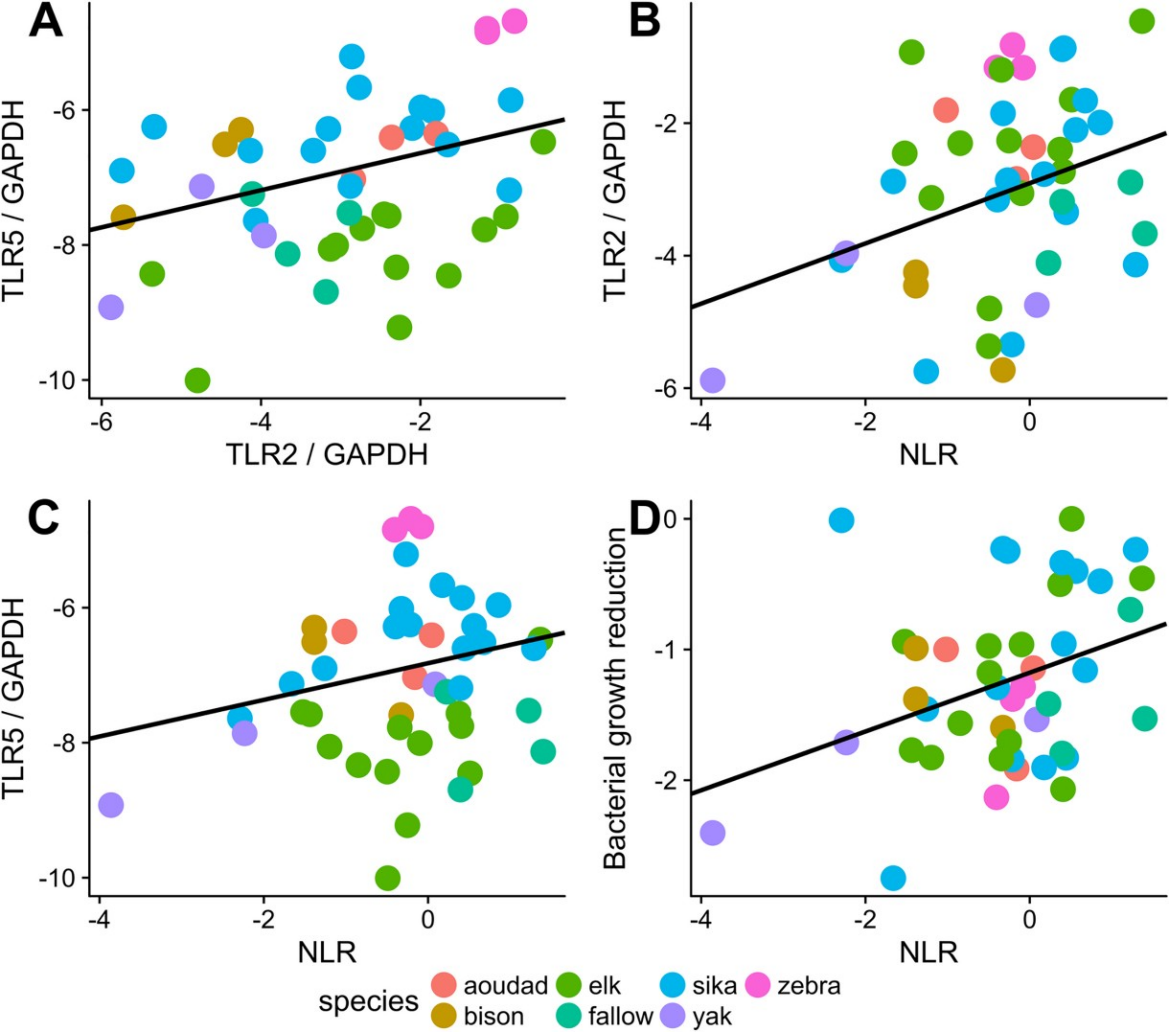
Measures of innate immunity were correlated in individual animals after accounting for species differences. Each data point represents an individual animal with log-transformed values and points color-coded by species. Correlations were found between neutrophil to lymphocyte ratio (NLR), toll-like receptors 2 and 5 (TLR2 and 5), and a plasma bacterial killing assay (BKA). BKA did not correlate with the TLRs. TLR2 and TLR5 were positively correlated (A, β = 0.744, SE = 0.216, p = 0.002, R_Σ_^2^ = 0.287). Additionally, the NLR positively correlated with TLR2 (B, β = 0.249, SE = 0.105, p = 0.023, R_Σ_^2^ = 0.113), TLR5 (C, β = 0.360, SE = 0.172, p = 0.044, R_Σ_^2^ = 0.129), and BKA (D, β = 0.497, SE = 0.218, p = 0.029, R_Σ_^2^ = 0.091).

We next determined if the varying innate immune parameters measured showed positive correlations with each other (Table 2). To do this, we constructed mixed linear models with each parameter held as the dependent variable. Because we did observe some variation in immune parameters between species (Fig 1), species was held as a random effect. We found positive correlation between the following immune measures: TLR2 and TLR5, NLR and TLR2, NLR and TLR5, and NLR and BKA (Fig 2). TLR2 and TLR5 were positively correlated (β = 0.744, SE = 0.216, p = 0.002, R_Σ_^2^ = 0.287). Additionally, the NLR positively correlated with TLR2 (β = 0.249, SE = 0.105, p = 0.023, R_Σ_^2^ = 0.113), TLR5 (β = 0.360, SE = 0.172, p = 0.044, R_Σ_^2^ = 0.129), and BKA (β = 0.497, SE = 0.218, p = 0.029, R_Σ_^2^ = 0.091). Sample sizes were too small to resolve correlations of immune measures within a species. If we include sex in the mixed linear model, sex is never a significant parameter in the outcomes and the previous relationships all remain significant.

**Table 2:** Summary of mixed-effect models comparing innate immunity measures. Linear mixed models (see Methods) were used to find correlations between neutrophil to lymphocyte ratio (NLR), toll-like receptors 2 and 5 (TLR2 and 5), and a plasma bacterial killing assay (BKA). All parameters were log-transformed. Species was used as a random effect. The estimates of dependent variables are displayed with standard error below in parentheses. Asterisks designate significance.

	*Dependent variable*:											
	TLR2			TLR5			NLR			BKA		
	(1)	(2)	(3)	(4)	(5)	(6)	(7)	(8)	(9)	(10)	(11)	(12)
**TLR5**	0.744***							0.360**				0.086
	**(0.216)**							**(0.172)**				(0.092)
**NLR**		0.455**			0.271**					0.225**		
		**(0.199)**			**(0.116)**					**(0.091)**		
**BKA**			0.446			0.223			0.497**			
			(0.301)			(0.173)			**(0.218)**			
**TLR2**				0.275***			0.249**				0.101	
				**(0.082)**			**(0.105)**				(0.068)	
**Constant**	2.081	-2.906***	-2.496***	-6.089***	-6.822***	-6.637***	0.360	2.076*	0.261	-1.177***	-0.968***	-0.668
	(1.563)	(0.428)	(0.625)	(0.458)	(0.447)	(0.504)	(0.416)	(1.246)	(0.401)	(0.099)	(0.230)	(0.659)
R_Σ_^2^	0.287	0.097	0.036	0.154	0.07	0.019	0.113	0.129	0.091	0.125	0.049	0.023

Note

*p<0.1

**p<0.05

***p<0.01

## Discussion

In this study, we describe comparisons of immune parameters related to innate defenses against bacterial pathogens across seven wild ungulate species. We found that different parameters of innate immune defense (TLR2 and TLR5 expression, NLR, and BKA) were correlated significantly and positively within individuals across our seven study-species and found clear differences in the innate immune mechanisms in the ungulates studied. Despite the lack of stimulation and chronic disease in our study animals, the data suggest that both TLRs tended to covary in ungulates. TLR2 stimulation may alter the expression of TLR5 as expression of TLR5 is upregulated in neutrophils from patients with cystic fibrosis in response to TLR2 stimulation [32]. Harada et al. [33] noted increased expression of both TLR2 and TLR5 in human biliary epithelial cells exposed to interferon gamma (IFNγ) while Homma et al. [34] found that IFNγ in combination with other stimulants upregulated TLR2 but not TLR5 in human respiratory epithelial cells, suggesting that there may be cell type specific expression patterns. TLR2 and TLR5 expression differences have been noted in macrophage-like cells as well: infection with *Borrelia burgdorferi* upregulated both TLR2 and TLR5 expression in microglial cells, but only TLR2 was upregulated in monocytes [35,36]. Exploring the interplay between simultaneous TLR2 and TLR5 expression would be an interesting future study.

Not only were TLR2 and TLR5 transcripts positively correlated with each other but also with NLR. Neutrophil abundance is often used as a measure of innate immune competence in comparative studies [6], and neutrophils are known to express TLRs, so it is perhaps not surprising that TLR transcript levels were elevated in the presence of increased NLR. Previously, it has been shown that neutrophil count is positively correlated to the ability of plasma to kill bacteria [13] or increase together in a specific treatment [27,37]; perhaps suggesting that coordinated antibacterial innate immune responses result in both higher levels of plasma proteins (i.e. complement) and cellular effector populations (i.e. neutrophils). Though we did not directly measure it, activation of the complement system can have a direct effect on neutrophils as the cleaved C3 and C5 proteins (C3a and C5a) act as anaphylatoxins and recruit neutrophils to the sites of activated complement [38]. Interestingly, while neutrophil abundance did not vary between species, the NLR did vary. Bovids tend to have higher lymphocyte counts than other species [39,40], and we do find elevated levels of lymphocytes in species such as yak and bison, which may explain the lower NLR in these animals. However, the NLR did positively correlate with BKA when species was held as a random effect in our model, suggesting that this relationship was not due to a species-specific effect. Summarizing the positive correlations of innate immune defenses, we find the TLR transcripts correlate with each other, and TLRs and BKA correlate with NLR. As such, our results document striking positive covariance between innate, antibacterial immune mechanisms.

A common objective in ecoimmunological studies is measuring an accurate and relevant snapshot of immune function in wild species. Studies must treat these often-simple measurements as proxies for the entire complex immune response [41] and interpret them in the context of ecological characteristics [42]. The assumption that observed immunological patterns are robust to the choice of immune measure is essential for interpreting and comparing trends across ecoimmunological studies but has rarely been tested. We found positive correlations between measures of innate immune surveillance (TLR2, 5), available effector cells (NLR), and functional antibacterial defense (BKA), validating the notion that different assays commonly used in ecoimmunological studies, are likely to yield similar patterns of interspecific variation in innate immunity.

Future work will need to be undertaken to determine if these broad patterns of concordance among innate, antibacterial immune responses occur in animals exposed to particular bacterial pathogens. While our data demonstrate positive correlations with innate immune surveillance and the killing of a laboratory strain of bacteria chosen for its reliability in this assay, we do not know if this will translate into increased protection against specific pathogenic bacteria. It will also be necessary to extend these findings into a field setting rather than a zoological one, as captivity may have profound effects on immune responses [43,44]. Finally, it will be important to determine if these patterns are routinely observed within individuals of a singular species, which will undoubtedly require increased sample sizes to fully appreciate. It is our hope that this work will encourage further research to compare across common ecoimmunological assays evaluating adaptive immune mechanisms and studies including a broader range of taxa.

## Supporting information

S1 Fig Representative TLR2 and TLR5 PCR products from all seven species tested. RNA from each indicated species was converted to cDNA and used as a template for PCR with primers designed to amplify TLR2 or TLR5. PCR products were resolved by gel electrophoresis on a 2% agarose gel and visualized using ethidium bromide to stain DNA. A DNA ladder with indicated sizes is shown.
(TIF)

S1 Table Primer sets for TLR quantitative PCR of seven ungulates at Wildlife Safari. (DOCX)

S2 Table Wildlife Safari study individuals. These ungulates were sampled 2013–2014. Health status indicates minor health problems of some animals included in the study.
(DOCX)

## References

[1] Medzhitov R, Janeway CA. Innate immunity: Impact on the adaptive immune response. Curr Opin Immunol. 1997;9: 4–9. 10.1016/s0952-7915(97)80152-5 9039775

[2] Gabay C, Kushner I. Mechanisms of disease: Acute-phase proteins and other systemic responses to inflammation. N Engl J Med. 1999;340: 448–454. 10.1056/NEJM199902113400607 9971870

[3] Kawai T, Akira S. The roles of TLRs, RLRs and NLRs in pathogen recognition. Int Immunol. 2009;21: 317–337. 10.1093/intimm/dxp017 19246554 PMC2721684

[4] Brubaker SW, Bonham KS, Zanoni I, Kagan JC. Innate Immune Pattern Recognition: A Cell Biological Perspective. Annual Review of Immunology Vol 33. 2015;33: 257–290. 10.1146/annurev-immunol-032414-112240 25581309 PMC5146691

[5] Silva MT, Correia-Neves M. Neutrophils and macrophages: the main partners of phagocyte cell systems. Front Immunol. 2012;3 10.3389/fimmu.2012.00174 22783254 PMC3389340

[6] Demas GE, Zysling DA, Beechler BR, Muehlenbein MP, French SS. Beyond phytohaemagglutinin: assessing vertebrate immune function across ecological contexts. J Anim Ecol. 2011;80: 710–730. 10.1111/j.1365-2656.2011.01813.x 21401591

[7] Downs CJ, Stewart KM. A primer in ecoimmunology and immunology for wildlife research and management. Calif Fish Game. 2014;100: 371–395.

[8] de Oliviera Nascimento L, Massari P, Wetzler L. The Role of TLR2 in Infection and Immunity. Front Immunol. 2012;3 10.3389/fimmu.2012.00079 22566960 PMC3342043

[9] Yoon SI, Kurnasov O, Natarajan V, Hong MS, Gudkov AV, Osterman AL, et al Structural Basis of TLR5-Flagellin Recognition and Signaling. Science. 2012;335: 859–864. 10.1126/science.1215584 22344444 PMC3406927

[10] Guthrie GJK, Charles KA, Roxburgh CSD, Horgan PG, McMillan DC, Clarke SJ. The systemic inflammation-based neutrophil-lymphocyte ratio: Experience in patients with cancer. Critical Reviews in Oncology Hematology. 2013;88: 218–230. 10.1016/j.critrevonc.2013.03.010 23602134

[11] Davis AK, Maney DL, Maerz JC. The use of leukocyte profiles to measure stress in vertebrates: a review for ecologists. Funct Ecol. 2008;22: 760–772.

[12] Fowler ME, Miller RE. Zoo and wild animal medicine. 5th ed St. Louis, Mo.: St. Louis, Mo.: Saunders; 2003.

[13] Beechler BR, Broughton H, Bell A, Ezenwa VO, Jolles AE. Innate Immunity in Free-Ranging African Buffalo (Syncerus caffer): Associations with Parasite Infection and White Blood Cell Counts. Physiol Biochem Zool. 2012;85: 255–264. 10.1086/665276 22494981

[14] Liebl AL, Martin LB. Simple quantification of blood and plasma antimicrobial capacity using spectrophotometry. Funct Ecol. 2009;23: 1091–1096.

[15] Matson KD, Tieleman BI, Klasing KC. Capture stress and the bactericidal competence of blood and plasma in five species of tropical birds. Physiol Biochem Zool. 2006;79: 556–564. 10.1086/501057 16691521

[16] Dugovich BS, Peel MJ, Palmer AL, Zielke RA, Sikora AE, Beechler BR, et al Detection of bacterial-reactive natural IgM antibodies in desert bighorn sheep populations. PLoS One. 2017;12: 15. 10.1371/journal.pone.0180415 PMC5491220 28662203

[17] Levy O. Antimicrobial proteins and peptides of blood: templates for novel antimicrobial agents. Blood. 2000;96: 2664–2672. 11023496

[18] Yang D, Biragyn A, Hoover DM, Lubkowski J, Oppenheim JJ. Multiple roles of antimicrobial defensins, cathelicidins, and eosinophil-derived neurotoxin in host defense. Annu Rev Immunol. 2004;22: 181–215. 10.1146/annurev.immunol.22.012703.104603 15032578

[19] Jolles AE, Beechler BR, Dolan BP. Beyond mice and men: environmental change, immunity and infections in wild ungulates. Parasite Immunol. 2015;37: 255–266. 10.1111/pim.12153 25354672 PMC4414670

[20] Jolles AE, Ezenwa VO. Ungulates as model systems for the study of disease processes in natural populations. J Mammal. 2015;96: 4–15. 10.1093/jmammal/gyu007 PMC7107476 32287382

[21] Martin C, Pastoret P-P, Brochier B, Humblet M-F, Saegerman C. A survey of the transmission of infectious diseases/infections between wild and domestic ungulates in Europe. Vet Res. 2011;42: 70 10.1186/1297-9716-42-70 21635726 PMC3152899

[22] Cases-Diaz E, Marco I, Lopez-Olvera JR, Mentaberre G, Serrano E, Lavin S. Effect of Acepromazine and Haloperidol in Male Iberian Ibex (Capra pyrenaica) Captured by Box-Trap. J Wildl Dis. 2012;48: 763–767. 10.7589/0090-3558-48.3.763 22740543

[23] Marco I, Lavin S. Effect of the method of capture on the haematology and blood chemistry of red deer (Cervus elaphus). Res Vet Sci. 1999;66: 81–84. 10.1053/rvsc.1998.0248 10208884

[24] Strobel S, Becker NI, Encarnacao JA. No short-term effect of handling and capture stress on immune responses of bats assessed by bacterial killing assay. Mamm Biol. 2015;80: 312–315. 10.1016/j.mambio.2015.02.005 PMC7091759 32218714

[25] Tieleman BI, Williams JB, Ricklefs RE, Klasing KC. Constitutive innate immunity is a component of the pace-of-life syndrome in tropical birds. Proceedings of the Royal Society B-Biological Sciences. 2005;272: 1715–1720. 10.1098/rspb.2005.3155 PMC1559858 16087427

[26] French SS, Neuman-Lee LA. Improved ex vivo method for microbiocidal activity across vertebrate species. Biol Open. bio.biologists.org; 2012;1: 482–487. 10.1242/bio.2012919 23213440 PMC3507210

[27] Gervasi SS, Hunt EG, Lowry M, Blaustein AR. Temporal patterns in immunity, infection load and disease susceptibility: understanding the drivers of host responses in the amphibian-chytrid fungus system. Funct Ecol. 2014;28: 569–578.

[28] Dolan BP, Fisher KM, Colvin ME, Benda SE, Peterson JT, Kent ML, et al Innate and adaptive immune responses in migrating spring-run adult chinook salmon, Oncorhynchus tshawytscha. Fish Shellfish Immunol. 2016;48: 136–144. 10.1016/j.fsi.2015.11.015 26581919

[29] Oksanen J, Kindt R, Legendre P, O’Hara B. The vegan package. researchgate.net. Available: https://www.researchgate.net/profile/Gavin_Simpson/publication/228339454_The_vegan_Package/links/0912f50be86bc29a7f000000/The-vegan-Package.pdf

[30] Bates D, Mächler M, Bolker B, Walker S. Fitting Linear Mixed-Effects Models Using lme4. Journal of Statistical Software, Articles. 2015;67: 1–48.

[31] Jaeger BC, Edwards LJ, Das K, Sen PK. An R-2 statistic for fixed effects in the generalized linear mixed model. J Appl Stat. Taylor & Francis; 2017;44: 1086–1105.

[32] Koller B, Kappler M, Latzin P, Gaggar A, Schreiner M, Takyar S, et al TLR expression on neutrophils at the pulmonary site of infection: TLR1/TLR2-mediated up-regulation of TLR5 expression in cystic fibrosis lung disease. Mian Yi Xue Za Zhi. 2008;181: 2753–2763. 10.4049/jimmunol.181.4.2753 18684966

[33] Harada K, Isse K, Nakanuma Y. Interferon gamma accelerates NF-kappa B activation of biliary epithelial cells induced by toll-like receptor and ligand interaction. J Clin Pathol. 2006;59: 184–190. 10.1136/jcp.2004.023507 16443736 PMC1860324

[34] Homma T, Kato A, Hashimoto N, Batchelor J, Yoshikawa M, Imai S, et al Corticosteroid and cytokines synergistically enhance toll-like receptor 2 expression in respiratory epithelial cells. Am J Respir Cell Mol Biol. 2004;31: 463–469. 10.1165/rcmb.2004-0161OC 15242847

[35] Bernardino ALF, Myers TA, Alvarez X, Hasegawa A, Philipp MT. Toll-like receptors: Insights into their possible role in the pathogenesis of Lyme neuroborreliosis. Infect Immun. 2008;76: 4385–4395. 10.1128/IAI.00394-08 18694963 PMC2546821

[36] Cabral ES, Gelderblom H, Hornung RL, Munson PJ, Martin R, Marques AR. Borrelia burgdorferi lipoprotein-mediated TLR2 stimulation causes the down-regulation of TLR5 in human monocytes. J Infect Dis. 2006;193: 849–859. 10.1086/500467 16479520

[37] Young HS, Dirzo R, Helgen KM, McCauley DJ, Nunn CL, Snyder P, et al Large wildlife removal drives immune defence increases in rodents. Funct Ecol. 2016;30: 799–807.

[38] Mak TW, Saunders ME, Jett BD. Primer to the Immune Response. Elsevier Science; 2013.

[39] Maeda S. Veterinary Immunology: An Introduction—by Ian R. Tizard. Vet Dermatol. 2009;20: 144–144.

[40] Roland L, Drillich M, Iwersen M. Hematology as a diagnostic tool in bovine medicine. J Vet Diagn Invest. 2014;26: 592–598. 10.1177/1040638714546490 25121728

[41] Martin LB II, Weil ZM, Nelson RJ. Refining approaches and diversifying directions in ecoimmunology. Integr Comp Biol. 2006;46: 1030–1039. 10.1093/icb/icl039 21672805

[42] Prall SP, Muehlenbein MP. Testosterone and Immune Function in Primates: A Brief Summary with Methodological Considerations. Int J Primatol. 2014;35: 805–824.

[43] Viney M, Riley EM. The Immunology of Wild Rodents: Current Status and Future Prospects. Front Immunol. 2017 11 14;8:1481 10.3389/fimmu.2017.01481 29184549 PMC5694458

[44] Fair PA, Schaefer AM, Houser DS, Bossart GD, Romano TA, Champagne CD, et al The environment as a driver of immune and endocrine responses in dolphins (Tursiops truncatus). PLoS One. 2017 5 3;12(5):e0176202 10.1371/journal.pone.0176202 28467830 PMC5415355

