# Correction: Multiple innate antibacterial immune defense elements are correlated in diverse ungulate species

**DOI:** 10.1371/journal.pone.0227322

**Published:** 2020-01-02

**Authors:** Brian S. Dugovich, Lucie L. Crane, Benji E. Alcantar, Brianna R. Beechler, Brian P. Dolan, Anna E. Jolles

The third author’s name is incorrect. The correct name is: Benji E. Alcantar. The correct citation is: Dugovich BS, Crane LL, Alcantar BE, Beechler BR, Dolan BP, Jolles AE (2019) Multiple innate antibacterial immune defense elements are correlated in diverse ungulate species. PLoS ONE 14(11): e0225579. https://doi.org/10.1371/journal.pone.0225579
