# Supplementary figures and images for: Multiple innate antibacterial immune defense elements are correlated in diverse ungulate species
Source: PLoS One. 2019 Nov 27;14(11):e0225579. doi: 10.1371/journal.pone.0225579 (PMC6881064; doi:10.1371/journal.pone.0225579)

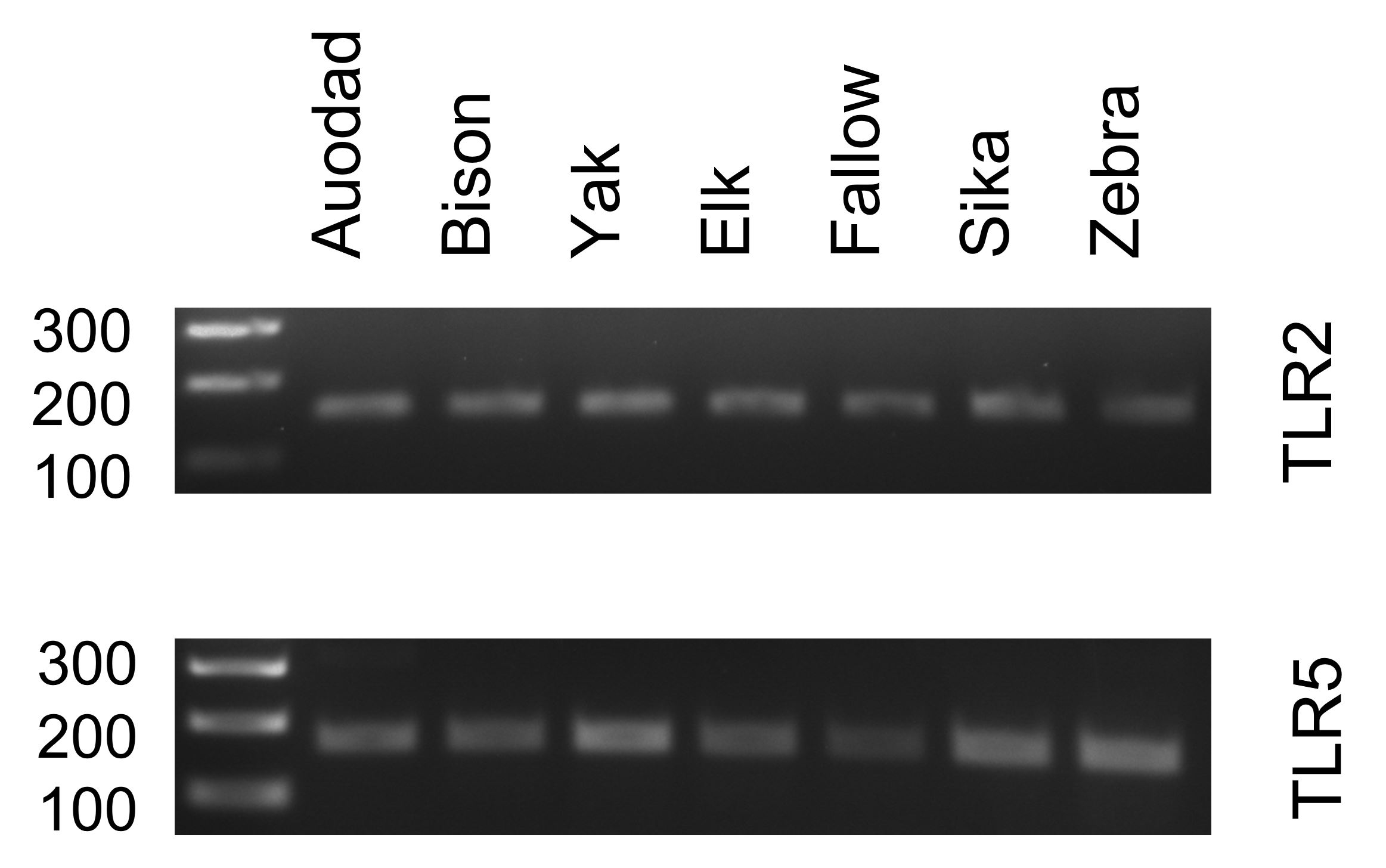

Supplement: S1 Fig — RNA from each indicated species was converted to cDNA and used as a template for PCR with primers designed to amplify TLR2 or TLR5. PCR products were resolved by gel electrophoresis on a 2% agarose gel and visualized using ethidium bromide to stain DNA. A DNA ladder with indicated sizes is shown. (TIF) [file pone.0225579.s001.tif]
