# Supplementary material for: Multiple innate antibacterial immune defense elements are correlated in diverse ungulate species
Source: PLoS One. 2019 Nov 27;14(11):e0225579. doi: 10.1371/journal.pone.0225579 (PMC6881064; doi:10.1371/journal.pone.0225579)
Supplement: S1 Table — (DOCX) [file pone.0225579.s002.docx]

| Target Gene | Primer Sequence (5’-3’) | Size of PCR product |
| --- | --- | --- |
| GAPDH | Forward - ATCAAGAAGGTRGTGAAGCAGG | 175 base pairs |
|  | Reverse - TGTCRTACCAGGAAATGAGCTT |  |
| TLR2 | Forward - ACKCTMCCRGATGCCTCCTT | 158 base pairs |
|  | Reverse - GACAGGAABTCACAGGAGCA |  |
| TLR5 | Forward - GACGCSTGGTGCCTSGAA | 170 base pairs |
|  | Reverse - TCYTCRGGCCACCTCAARTAC |  |
