# Supplementary material for: Multiple innate antibacterial immune defense elements are correlated in diverse ungulate species
Source: PLoS One. 2019 Nov 27;14(11):e0225579. doi: 10.1371/journal.pone.0225579 (PMC6881064; doi:10.1371/journal.pone.0225579)
Supplement: S2 Table — These ungulates were sampled 2013–2014. Health status indicates minor health problems of some animals included in the study. (DOCX) [file pone.0225579.s003.docx]

| Individual | Species | Sex* | Age (months) | Health Status |
| --- | --- | --- | --- | --- |
| WSP13-003 | elk | cm | 156.5 | ulcerated cornea |
| WSP13-004 | elk | cm | 144.7 | epistaxis |
| WSP13-005 | elk | f | 157.1 | normal |
| WSP13-006 | sika | f | 144.4 | normal |
| WSP13-013 | sika | f | 181.3 | normal |
| WSP13-015 | sika | f | 146 | hoof ulcer |
| WSP13-016 | aoudad | m | 147.1 | pododermatitis |
| WSP13-017 | sika | f | 133 | normal |
| WSP13-018 | fallow | f | 145.3 | abnormal eye |
| WSP13-020 | sika | f | 241.2 | normal |
| WSP13-027 | bison | f | 127.2 | normal |
| WSP13-028 | zebra | m | 19.6 | normal |
| WSP13-029 | elk | f | 165.4 | normal |
| WSP13-031 | sika | f | 133.6 | normal |
| WSP13-033 | yak | f | 143.5 | normal |
| WSP13-034 | elk | f | 146.9 | pododermatitis |
| WSP13-035 | aoudad | f | 87.7 | normal |
| WSP13-036 | bison | f | 43.8 | normal |
| WSP13-037 | zebra | f | 36.9 | capture hyperthermia |
| WSP13-038 | yak | f | 160.7 | normal |
| WSP13-039 | sika | f | 147 | normal |
| WSP13-040 | sika | m | 153.7 | overgrown hooves |
| WSP13-041 | aoudad | f | 74.7 | normal |
| WSP13-042 | zebra | f | 26.3 | normal |
| WSP13-043 | bison | f | 104.7 | normal |
| WSP13-044 | yak | f | 313.8 | lethargic |
| WSP14-01 | elk | f | 168.7 | normal |
| WSP14-02 | sika | f | 176.9 | normal |
| WSP14-03 | elk | f | 156.8 | normal |
| WSP14-04 | sika | f | 133 | normal |
| WSP14-05 | sika | f | 168 | normal |
| WSP14-06 | fallow | f | 190.5 | multiple masses |
| WSP14-07 | sika | f | 217.5 | normal |
| WSP14-08 | fallow | f | 157.1 | multiple masses |
| WSP14-09 | elk | f | 178.6 | normal |
| WSP14-10 | sika | f | 167.6 | normal |
| WSP14-11 | sika | f | 181.7 | normal |
| WSP14-12 | fallow | f | 156.3 | normal |
| WSP14-13 | sika | f | 192.9 | normal |
| WSP14-14 | elk | f | 167.9 | normal |
| WSP14-15 | elk | cm | 204.9 | minor laceration |
| WSP14-16 | elk | f | 181.6 | normal |
| WSP14-18 | elk | m | 158.2 | normal |
| WSP14-19 | elk | m | 30.9 | normal |

*cm refers to castrated males
